# Please Welcome Our First Academic Editor-in-Chief

**DOI:** 10.1371/journal.pbio.0060049

**Published:** 2008-02-26

**Authors:** Theodora Bloom, Jami Dantzker, Christine Ferguson, Liza Gross, Catriona MacCallum, Mark Patterson, Robert Shields, Janelle Weaver, Elizabeth Whiteman

## Abstract

The PLoS Biology Editors explain the background to Jonathan Eisen's appointment as the journal's first Academic Editor-in-Chief.

*PLoS Biology* was launched in 2003 with the goal of creating an open-access alternative to the very best subscription journals—a home for great science, which would be made available immediately and freely to any interested reader. We've come a long way toward achieving this goal, and the support of the scientific community has been paramount in the impressive standing that *PLoS Biology* has achieved in its short life. Nowhere can this be more clearly seen than in the hard-working *PLoS Biology* Editorial Board, whose consistent effort has brought us to where we are today.

At its inception, *PLoS Biology* deliberately created an editorial model that encouraged active participation of the Editorial Board. Although the editorial responsibility for *PLoS Biology* rests with the professional editors, we frequently consult with Academic Editors, usually drawn from the Editorial Board, to make decisions on manuscripts, and every published manuscript has been seen and commented on by an Academic Editor. In recognition of their contribution, the name of the Academic Editor is indicated on the final published article. It's an editorial model that makes *PLoS Biology* different from other top-tier journals, and one that many authors have indicated that they strongly support.

More than four years on, the journal and the Board have grown, and it has become increasingly challenging to communicate effectively with such a large (greater than 130 members) and diverse (“molecules to ecosystems”) Board and to provide the support that Board members need to become proactive, determined advocates for both *PLoS Biology* and open access. We have considered how best to respond to this challenge and how to make the most of our unique relationship with the academic community, and we naturally settled on the idea of asking a member of our Editorial Board to take on a leadership role as an Academic Editor-in-Chief.

While the responsibility for the content of *PLoS Biology* and for the editorial decision-making remains with the *PLoS Biology* professional editors, we envisaged that an Academic Editor-in-Chief would be a community representative for the journal both inside and outside of PLoS, and would work with the staff editors to help shape the future of the journal.

In the Academic Editor-in-Chief, we were looking for someone who has unequivocal commitment to open access, boundless energy, and the vision and foresight to help transform scientific discourse from the confines of traditional publishing into a world that takes full advantage of the online medium.

In considering potential candidates for the Academic Editor-in-Chief, one person in particular stood out. Jonathan Eisen has become a passionate open access spokesperson and advocate, and he now publishes his work only in open-access journals (see also the accompanying Editorial, doi:10.1371/journal.pbio.0060048, for more about Jonathan's reasons for his commitment to open access). Jonathan is a Professor at the University of California Davis Genome Center where he studies the evolution of novelty in microorganisms (see http://128.120.136.15/mediawiki/index.php/Main_Page), and he is an editorial advisor for both *PLoS Biology* and *PLoS Computational Biology*. We are thrilled that Jonathan has agreed to collaborate with PLoS as the Academic Editor-in-Chief for *PLoS Biology*, and we are looking forward to working with him in this new capacity.

